# Human Breast Cancer Cells Are Redirected to Mammary Epithelial Cells upon Interaction with the Regenerating Mammary Gland Microenvironment In-Vivo

**DOI:** 10.1371/journal.pone.0049221

**Published:** 2012-11-14

**Authors:** Karen M. Bussard, Gilbert H. Smith

**Affiliations:** Mammary Biology and Tumorigenesis Laboratory, National Cancer Institute, National Institutes of Health, Bethesda, Maryland, United States of America; Huntsman Cancer Institute, University of Utah, United States of America

## Abstract

Breast cancer is the second leading cause of cancer deaths in the United States. At present, the etiology of breast cancer is unknown; however the possibility of a distinct cell of origin, i.e. a cancer stem cell, is a heavily investigated area of research. Influencing signals from the tissue niche are known to affect stem cells. Literature has shown that cancer cells lose their tumorigenic potential and display ‘normal’ behavior when placed into ‘normal’ ontogenic environments. Therefore, it may be the case that the tissue microenvironment is able to generate signals to redirect cancer cell fate. Previously, we showed that pluripotent human embryonal carcinoma cells could be redirected by the regenerating mammary gland microenvironment to contribute epithelial progeny for ‘normal’ gland development in-vivo. Here, we show that that human metastatic, non-metastatic, and metastasis-suppressed breast cancer cells proliferate and contribute to normal mammary gland development in-vivo *without* tumor formation. Immunochemistry for human-specific mitochondria, keratin 8 and 14, as well as human-specific milk proteins (alpha-lactalbumin, impregnated transplant hosts) confirmed the presence of human cell progeny. Features consistent with normal mammary gland development as seen in intact hosts (duct, lumen formation, development of secretory acini) were recapitulated in both primary and secondary outgrowths from chimeric implants. These results suggest the dominance of the tissue microenvironment over cancer cell fate. This work demonstrates that cultured human breast cancer cells (metastatic and non-metastatic) respond developmentally to signals generated by the mouse mammary gland microenvironment during gland regeneration in-vivo.

## Introduction

Approximately one in eight women in the United States will develop breast cancer during her lifetime [1]. The tissue microenvironment, in particular, is known to play a pivotal role in breast cancer initiation, progression, and regulate the malignant phenotype of tumors [2]. The “cancer cell reversibility” concept was explored by Mintz and Illmensee whom inoculated OTT 6050 ascites teratoma cells either subcutaneously into mice or into blastocysts that were subsequently implanted into pseudopregnant hosts [3]. Teratocarcinomas formed in mice directly inoculated with teratoma cells. However, when the teratoma cells were inoculated into ‘normal’ ontogenic environments (blastocysts), the cancer cells lost their tumorigenic potential and displayed ‘normal’ behavior [3], [4]. These studies suggest that cancer cells may be restored to ‘normal’ function in the appropriate tissue microenvironment. More recently, Felsher et al. discovered that in order to become tumorigenic, an oncogene must be in an environment permissive for tumor development [5]. Thus, if conditions did not favor tumorigenesis, no tumor would grow. Further, Hochedlinger et al. utilized nuclear transplantation to introduce nuclei from malignant cancer cells into enucleated oocytes, which subsequently were used to produce chimeric mice [6]. Even though the mice had a predisposition for a tumorigenic phenotype, the majority of their tissues were normal; regulated by the ‘normal’, non-tumorigenic microenvironment of the enucleated oocyte [6].

Recently, we showed that NTERA2 (NT2) human embryonal carcinoma cells could be redirected from their tumorigenic phenotype to differentiation into *functional* bona fide human-specific mammary epithelial cells through interaction with the mouse mammary microenvironment in-vivo [7]. NT2 cells, however, are known to be pluripotent as well as cancer-producing (teratoma) cells [8]. In this study we test whether more committed human cancer cells, in this case, human breast cancer cells would be re-directed to normal breast epithelial states by interaction with regenerating mouse mammary cells in-vivo. Therefore, we hypothesized that the mammary gland microenvironment may be capable of generating signals to normalize human breast cancer cells to a non-tumorigenic cell fate in-vivo. To test this hypothesis, human MDA-MB-231-GFP metastatic, MDA-MB-231BRMS-GFP metastasis-suppressed, and MDA-MB-468 non-metastatic breast cancer cells were mixed with mouse mammary epithelial cells and inoculated into mammary fat pads of mice cleared of epithelium. It was found that human metastatic, non-metastatic, and metastasis-suppressed breast cancer cells proliferate and contribute to normal mammary gland development *without* tumor formation through interaction with the regenerating mouse mammary microenvironment in-vivo. In addition, human breast cancer cells expressed human mammary-specific luminal and myoepithelial keratins and exhibited no evidence of cell-cell fusion in the chimera. These results suggest the dominance of the “normal” tissue microenvironment over cancer cell fate.

## Materials and Methods

### Mice

Three-week-old female athymic nude mice were used as hosts for transplantation studies. All mice were housed in Association for Assessment and Accreditation of Laboratory Animal Care-accredited facilities in accordance with the National Institutes of Health Guide for the Care and Use of Laboratory Animals. The National Cancer Institute Animal Care and use Committee approved all experimental procedures.

### Cells

MDA-MB-231-GFP human metastatic breast cancer cells were derived from a pleural effusion of an adenocarcinoma engineered to express GFP [9]. MDA-MB-231BRMS-GFP cells have been engineered to express GFP and are metastasis-suppressed to bone and other organs by transfection of the BRMS1 gene [10], [11]. MDA-MB-468 human non-metastatic breast cancer cells were derived from the pleural effusion of a metastatic breast adenocarcinoma [9]. All cells were a gift from Dr. Danny Welch, Kansas University Medical Center. Cells were maintained in DMEM (Invitrogen, Carlsbad, CA), 5% FBS (Invitrogen), and penicillin 100 U/ml/streptomycin 100 µg/ml (Invitrogen). Cells were cultured in a humidified chamber of 5% CO_2_ and 95% air at 37°C.

hTERT-HME1 human mammary epithelial cells were obtained from normal breast tissue and immortalized by infection with retrovirus pBabepuro+hTERT vector [12], [13]. These cells were a gift from Dr. Henry Donahue, The Pennsylvania State University, Hershey, PA. hTERT-HME1 cells were maintained in Clonetics® MEGM® Mammary Epithelial Cell Growth Medium supplemented with bovine pituitary extract, GA-1000, human epidermal growth factor, insulin, and hydrocortisone (Lonza, Walkersville, MD). Cells were cultured in a humidified chamber of 5% CO_2_ and 95% air at 37°C.

Mammary epithelial cells were collected from the mammary glands of 10–12 week old Balb/C female mice. Following dissociation in collagenase (1 mg/ml, Sigma, St. Louis, MO), cells were grown for 4 to 7 days as primary cultures on plastic culture flasks in DMEM (Invitrogen), 10% FBS (Invitrogen), insulin (1.0 µg/ml, Invitrogen), and epidermal growth factor (10.0 ng/ml, Invitrogen). Fibroblasts were reduced before collection of the epithelial cells via differential trypsinization [14].

### Cell and tissue transplantation

Human breast cancer cells were mixed with mouse mammary epithelial cells at ratios of 10,000 human breast cancer cells : 50,000 mouse mammary epithelial cells (MDA-MB-231-GFP, CD44-enriched MDA-MB-231-GFP, or CD44-depleted MDA-MB-231-GFP) or 1,000 human breast cancer cells : 50,000 mouse mammary epithelial cells (MDA-MB-231BRMS-GFP; MDA-MB-468). As a negative control, 10,000 hTERT-HME1 human mammary epithelial cells were mixed with 50,000 mouse mammary epithelial cells. (When 10,000 MDA-MB-231BRMS-GFP or 10,000 MDA-MB-468 human breast cancer cells mixed with 50,000 mouse mammary epithelial cells, mammary tumors formed 100% of the time (Table S1). Therefore, we elected to conduct subsequent experiments using 1,000 MDA-MB-231BRMS-GFP, 1,000 MDA-MB-468, or 10,000 MDA-MB-231 human breast cancer cells *plus* 50,000 mouse mammary epithelial cells to avoid complications resulting from tumor formation (Table S1)). Cell mixtures were resuspended in 10 µl and injected into the epithelium-divested abdominal fat pads of 3-week-old female athymic Nu/Nu mice. MDA-MB-231-GFP, MDA-MB-231BRMS-GFP, and MDA-MB-468 human breast cancer cells (positive control; 1,000 or 10,000 cells) or hTERT-HME1 cells (negative control; 10,000 cells) were inoculated alone as controls. Twelve weeks later, mice were mated or maintained as virgins, then subsequently euthanized. Fat pad outgrowths were harvested and either fragments cut for re-implantation as secondary outgrowths, dissociated in collagenase and prepared as primary tissue cultures, or examined after sectioning paraffin embedded whole mounts through immunostaining.

### Whole mount preparation

Abdominal mammary gland outgrowths were excised, spread on glass slides, and fixed in Carnoy's fixative overnight. Axillary glands from the host mice served as intact controls. Glands were subsequently stained with Carmine Alum at room temperature for 4–6 hours, followed by dehydration in a series of graded alcohols and xylene to remove fatty stroma [15].

### Immunochemistry

Coverslips were removed from paraffin embedded whole mounts using xylene. Whole mounts were rehydrated through a series of graded alcohols, embedded in paraffin and cut into 6 µm sections for staining. Primary antibodies used included rabbit polyclonal anti-human keratin 8 (1∶100; Abcam, Cambridge, MA), rabbit polyclonal anti-human keratin 14 (1∶100; Abcam), anti-mouse keratin 14 (1∶100, a gift from D. Roop; Baylor College of Medicine, Houston, Texas) [16], mouse monoclonal anti-human mitochondria (1∶50; PhosphoSolutions, Aurora, CO), anti-mouse total caseins (1∶500, rabbit polyclonal) [17], and goat polyclonal anti-human alpha-lactalbumin (1∶100; Santa Cruz, Santa Cruz, CA). For detection of GFP, rabbit polyclonal-GFP antibody from Invitrogen was used at 1∶25. This antibody had been tested earlier to detect MDA-MB-231-GFP and MDA-MB-231BRMS-GFP cells in bone metastases [18] and was found to be specific and effective for detection of GFP expression in histological sections. Sections were permeabilized for 10 minutes at room temperature using 0.2% Triton-X (Sigma) in phosphate buffered saline (PBS), followed by antigen retrieval in boiling citrate buffer (10 mmol/l; pH 6.0) for 3.5 minutes. Endogenous peroxidase activity was blocked with PEROXIDAZED (Biocare Medical, Concord, CA) for 20 min at room temperature and non-specific binding was blocked with either 10% goat serum (Sigma) or 10% rabbit serum (anti-human alpha-lactalbumin, SouthernBiotech, Birmingham, AL) in TBS-X (Sigma) for 1 hour. Sections were incubated with primary antibodies overnight at 4°C. For sections stained for human keratin 8, 14, mitochondria, alpha-lactalbumin or mouse keratin 14 or casein, sections were incubated with either rhodamine (1∶3500; Abcam), FITC (1∶3000; Abcam), or Alexa Fluor® 488 (1∶100; Invitrogen) for one hour at room temperature, followed by staining with 4′, 6-diamidino-2-phenylindole (DAPI) (0.2 ng/µl) for 4 minutes. The remaining sections were stained following the protocol supplied from Vector Laboratories for the R.T.U. Vectastain® Universal Elite ABC kit (Vector Laboratories, Burlingame, CA), DAB Substrate kit for peroxidase (Vector Laboratories), and counterstained using Gill's Hematoxylin (Sigma). Sections were mounted using either VectaMount (DAB sections; Vector Laboratories) or Fluoromount-G (Fluorescent sections; SouthernBiotech). Sections without the primary antibody, untreated human, and untreated mouse tissue served as controls. Images were viewed using a Zeiss Axioskop 2 plus (Carl Zeiss Microscopy, LLC. Thornwood, NY).

### Metaphase spreads

Human breast cancer cells and hTERT-HME1 cells pre-implantation, and cells made from primary cultures of harvested chimeric (human breast cancer cells in mouse mammary gland) secondary outgrowths were split 24 hours prior use. Next, cells were treated with 0.1 µg/ml colcemid (Invitrogen) for 6–24 hours and resuspended in 0.075M KCl warmed to 37°C for 20 minutes. Cells were fixed in a 3∶1 mixture of methanol and acetic acid and dropped ∼50 cm onto glass slides. Metaphase spreads were stained with DAPI (0.2 ng/µl) and visualized using a Zeiss Axioskop 2 plus or prepared for FISH chromosome painting.

### Fluorescence in-situ hybridization (FISH)

Mouse and human chromosome X specific painting probes were generated by bivariate chromosome flow sorting and were labeled subsequently by degenerate oligonucleotide primed PCR amplification (DOP-PCR) using Spectrum Green and Spectrum Orange (Abbott Molecular Inc, Abbott Park, IL) [19], [20]. In-situ hybridizations of the mouse or human probes were performed with 300–400 ng of PCR product per probe with 10 µg of mouse or human Cot-1 precipitated, dissolved in 10 µl hybridization buffer (formamide 50%, dextran sulfate 10%, 2× SSC), denatured at 80°C for 5 minutes, and reannealed at 37°C for 90 minutes before hybridization. Metaphase slides were denatured in 70% formamide/2× SSC, at 65°C for 80 seconds, and quenched in ice-cold 70% ethanol followed by dehydration at room temperature through a 70%, 90%, and 100% ethanol series. Probes were hybridized overnight in a humidity chamber at 37°C. Slides were washed, counterstained with DAPI (0.8 ng/µl) for 10 minutes, and mounted with antifade. Analyses were performed under an Axioplan 2 (Carl Zeiss Microscopy, LLC) fluorescence microscope coupled with a CCD camera (Photometrics, Tucson, AZ), and images captured with FISHview 4.5 software (Applied Spectral Imaging Inc., Vista, CA).

### Magnetic cell sorting

Cells made from primary cultures of harvested chimeric outgrowths were enumerated, and resuspended in a buffer containing PBS, pH 7.2, 0.5% bovine serum albumin, and 2 mM ethylene-diamine tetra-acetic acid (EDTA). Cells were then incubated for 15 minutes at 4°C with anti-human CD44 MicroBeads (Miltenyi Biotec, Auburn, CA), washed and resuspended in buffer, transferred to a prewetted LS MACS separation column (Miltenyi Biotec), and allowed to elute. Following two washes and elution of the negative fraction, the column was removed from the magnet, and human CD44 positive fraction collected. A second elution was carried out to increase positive fraction recovery.

### Tumorsphere culture

Human breast cancer cells pre-implantation, CD44-enriched human breast cancer cells pre-implantation, and CD44-depleted human breast cancer cells pre-implantation were enumerated and washed with PBS to remove serum. Following resuspension as a single cells in DMEM (Invitrogen), 40 ng/ml basic fibroblast growth factor (Invitrogen), 20 ng/ml human epidermal growth factor (Invitrogen), 4 µg/ml heparin (Sigma), and 2% B27 (Invitrogen), approximately 4,000 cells/well were plated in ultra-low attachment 6-well plates (Corning, Lowell, MA). Tumorspheres of at least 50 µm in diameter formed 7–10 days later and were identified by the appearance of a prominent extracellular membrane with no presence of individual cells. Tumorspheres were propagated via enzymatic dissociation by incubation in 0.25% trypsin-EDTA (Invitrogen) for 3 minutes at 37°C [21].

## Results

### Metastatic, non-metastatic, and metastasis-suppressed cells contribute to the formation of a differentiated and non-tumor-containing mammary gland

**Figure 1:**
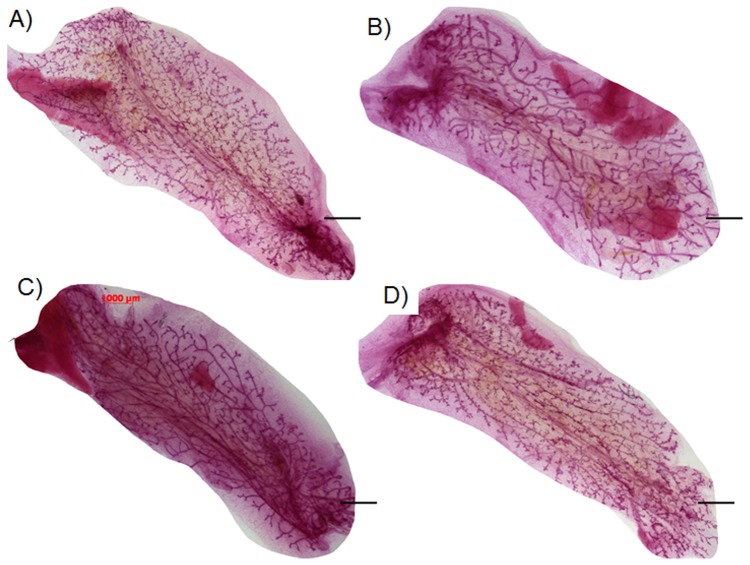
Human breast cancer cells differentiate in the mouse mammary gland. Whole mounts of chimeric mammary gland outgrowths (A–D) were formed from the implantation of fragments from first generation chimeric mammary gland outgrowths (Figure S1A–D). Twelve weeks post implantation, these secondary chimeric mammary gland outgrowths were harvested, fixed in Carnoy's fixative, and stained overnight with Carmine Alum. A) Second generation whole mount of chimeric mammary gland outgrowth formed from implantation of a fragment from an original MDA-MB-468 chimera (1 K MDA-MB-468 human non-metastatic breast cancer cells plus 50 K primary mouse mammary epithelial cells); B) Second generation whole mount of chimeric mammary gland outgrowth formed from implantation of a fragment from an original MDA-MB-231-GFP chimera (10 K MDA-MB-231-GFP human metastatic breast cancer cells plus 50 K primary mouse mammary epithelial cells); C) Second generation whole mount of chimeric mammary gland outgrowth formed from implantation of a fragment from an original MDA-MB-231BRMS-GFP chimera (1 K MDA-MB-231BRMS-GFP metastasis-suppressed breast cancer cells plus 50 K primary mouse mammary epithelial cells); D) Second generation whole mount of chimeric mammary gland outgrowth formed from implantation of a fragment from an original hTERT-HME1 chimera (10 K hTERT-HME1 human mammary epithelial cells plus 50 K mouse mammary epithelial cells). Scale bars 1000 µm.

**Figure 2:**
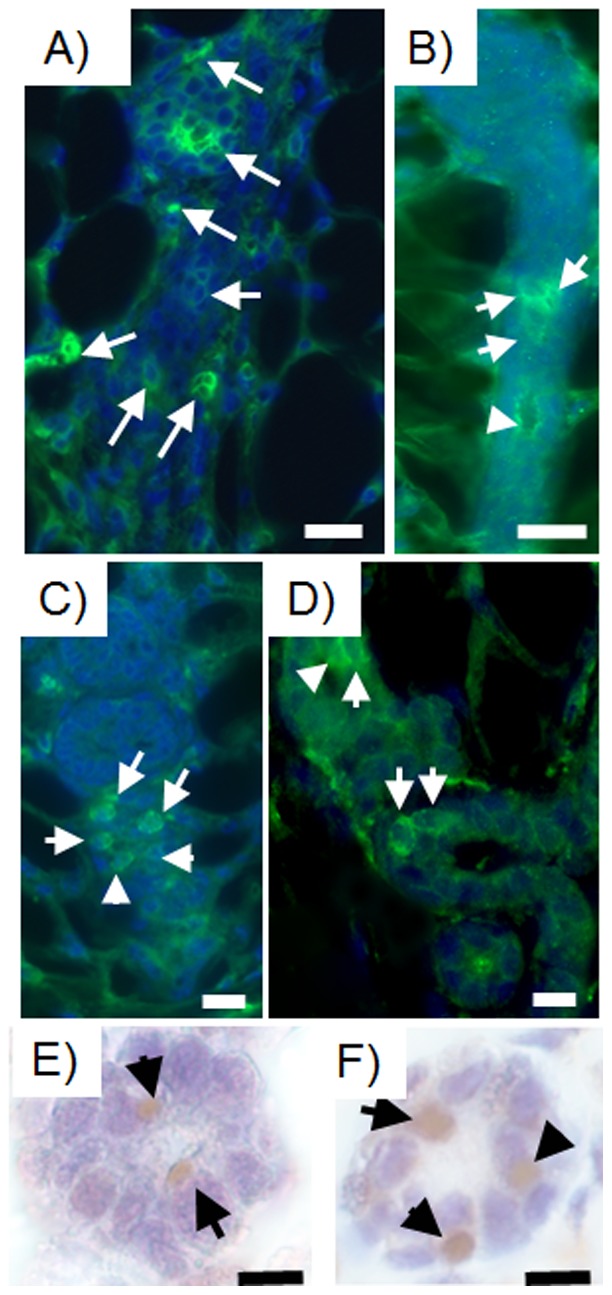
Human breast cancer cells are present in chimeric mammary outgrowths. Human breast cancer cells incorporated into the mammary gland and express human-specific mitochondria as indicated by arrows (A–D) (green; Alexa Fluor 488) and green fluorescent protein as demonstrated by immunoperoxidase (E–F) (brown, DAB). Human specific mitochondria is expressed in chimeric mammary gland outgrowths produced with A) MDA-MB-468 plus mammary epithelial cells transplant fragment; B) MDA-MB-231-GFP plus mammary epithelial cells transplant fragment; C) MDA-MB-231BRMS-GFP plus mammary epithelial cells transplant fragment; D) hTERT-HME1 plus mammary epithelial cells transplant fragment. Green fluorescent protein (GFP) as demonstrated by immunoperoxidase staining is expressed in chimeric mammary gland outgrowths generated using E) MDA-MB-231-GFP plus mammary epithelial cells transplant fragment; F) MDA-MB-231BRMS-GFP plus mammary epithelial cells transplant fragment. All outgrowths are second generation chimera transplants. Scale bars 40 µm.

Human MDA-MB-468 non-metastatic (1,000) [9], MDA-MB-231BRMS-GFP metastasis-suppressed (1,000) [10], [11], and MDA-MB-231-GFP (10,000) [9] metastatic breast cancer cells contributed to the formation of chimeric (human and mouse cells) outgrowths when implanted with mammary epithelial cells (50,000) in the epithelium divested fat pads of female athymic nude mice (Figure 1, Figure S1). The whole mounts pictured are the actual tissue outgrowths that were used for immunodetection of mouse and human cytokeratins, human mitochondria, and GFP. Chimeric outgrowths were found in ≥50% first and 70–100% second transplant generations (Table S1). Human cells were identified via immunohistochemistry in 100% of outgrowths sectioned (Table S1). No tumors formed in any of the chimeric outgrowths containing either 1,000 MDA-MB-468 non-metastatic human breast cancer, 1,000 MDA-MB-231BRMS metastasis-suppressed human breast cancer, 10,000 MDA-MB-231-GFP human breast cancer, or 10,000 hTERT-HME1 human mammary epithelial cells [12], [13] ***plus*** 50,000 mammary epithelial cells (Table S1). On the other hand, 10,000 MDA-MB-468 and 10,000 MDA-MB-231BRMS-GFP breast cancer cells inoculated with 50,000 mouse mammary epithelial cells produced tumors in 100% (5/5) of the inoculated fat pads (Table S1). In the case of the latter, the tumors were positive for GFP expression. Cells positive for human mitochondria (Figure 2a–d, Figure S2) also integrated into mammary ducts and lobules. We attempted to assay for human breast cancer cell integration in the chimeric glands using GFP fluorescence, however the fluorescence was undetectable at twelve weeks post-implantation in-vivo. Therefore, we assayed for GFP staining by immunoperoxidase and found that cells in mammary epithelial structures were positive for GFP staining in secondary outgrowths from the original chimeras (MDA-MB-231-GFP, MDA-MB-231BRMS-GFP, Figure 2e–f).

### Human breast cancer cells contribute to the formation of both luminal and myoepithelial cells in the chimeric mammary gland

**Figure 3:**
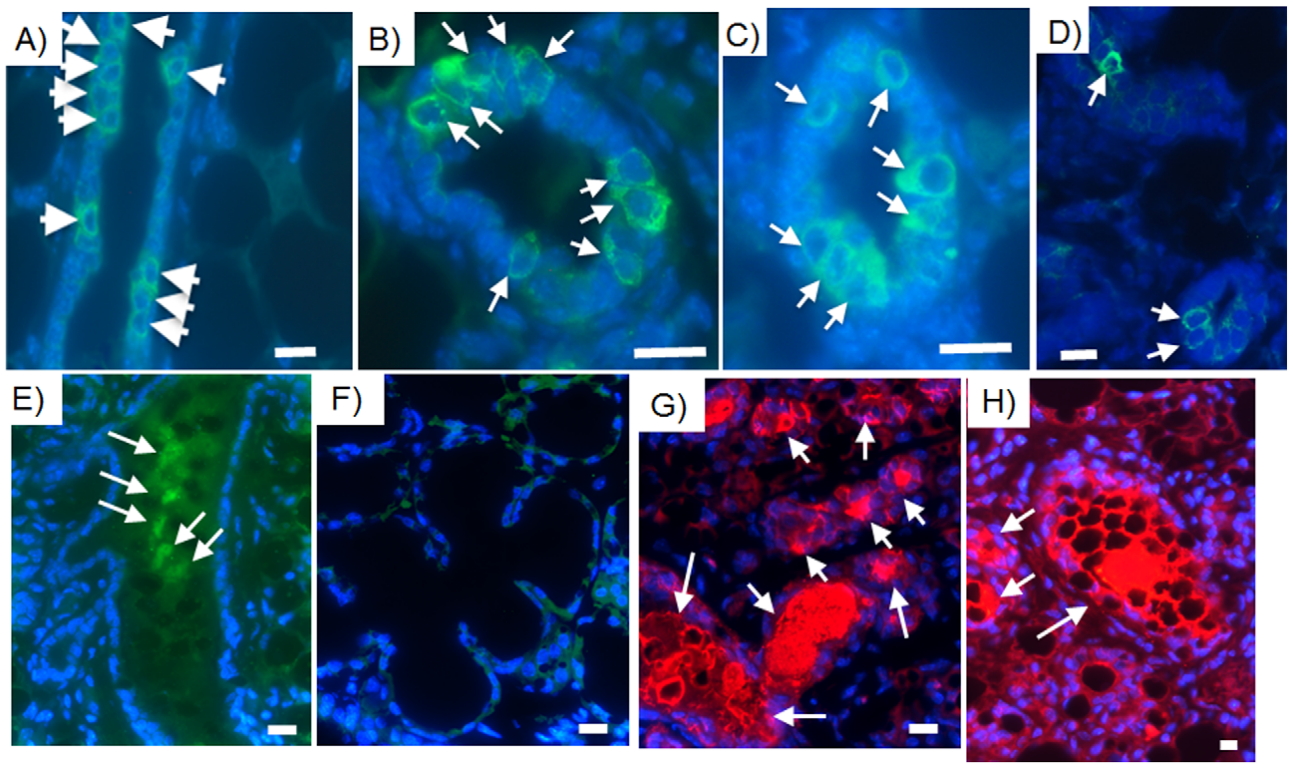
Human breast cancer cells contribute to the formation of luminal epithelial cells and secrete human milk proteins in the chimeric mammary gland. Human breast cancer cells incorporated into the mammary gland express human-specific luminal epithelial cell marker keratin 8 as indicated by the white arrows (green; Alexa Fluor 488) and secrete milk proteins in impregnated hosts (arrows, human alpha-lactalbumin, green, FITC; mouse casein, red, rhodamine). Human-specific luminal epithelial cell marker keratin 8 (arrows) is expressed in chimeric mammary gland outgrowths produced with A) MDA-MB-468 plus mammary epithelial cells transplant fragment, B) MDA-MB-231-GFP plus mammary epithelial cells transplant fragment; C) MDA-MB-231BRMS-GFP plus mammary epithelial cells transplant fragment; D) hTERT-HME1 human epithelial cells plus mammary epithelial cells transplant fragment. Anti-human alpha-lactalbumin (arrows) is expressed in chimeric mammary gland outgrowths generated using E) MDA-MB-231BRMS-GFP plus mammary epithelial cells transplant fragment; F) intact host. Anti-mouse caseins (arrows) is expressed in chimeric mammary gland outgrowths generated using G) MDA-MB-231-GFP plus mammary epithelial cells transplant fragment; H) intact host. All outgrowths are second generation chimeras (human breast cancer cells plus mouse mammary epithelial cells). Intact glands are from lactating hosts. Scale bars 10 µm.

**Figure 4:**
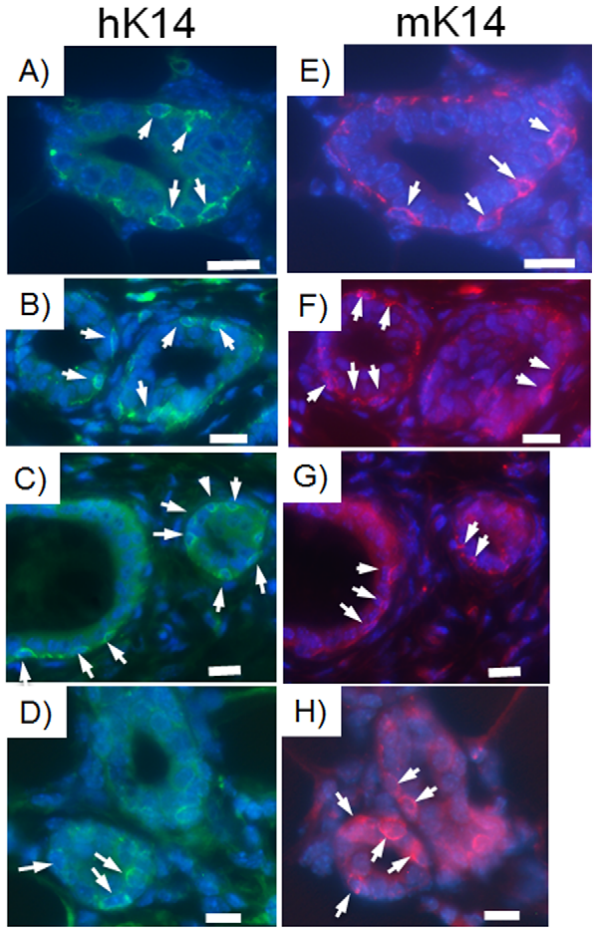
Human keratin 14 and mouse keratin 14 are expressed in basal cells of consecutive sections of the same second generation chimeric duct. Human breast cancer cells contribute to the formation of basal cellular structures via the expression of the myoepithelial cell marker keratin 14 in the mouse mammary gland. Human-specific myoepithelial cell marker keratin 14 (arrows) is expressed in chimeric mammary gland outgrowths produced with A, E) MDA-MB-468 plus mammary epithelial cell transplant fragment; B, F) MDA-MB-231-GFP plus mammary epithelial cell transplant fragment; C,G) MDA-MB-231BRMS-GFP plus mammary epithelial cell transplant fragment; D,H) hTERT-HME1 plus mammary epithelial cell transplant fragment. A–D) Human-specific keratin 14; E–H) mouse-specific keratin 14. All outgrowths are second generation chimeras (human breast cancer/human mammary epithelial cells plus mouse mammary epithelial cells). Scale bars 10 µm.

**Figure 5:**
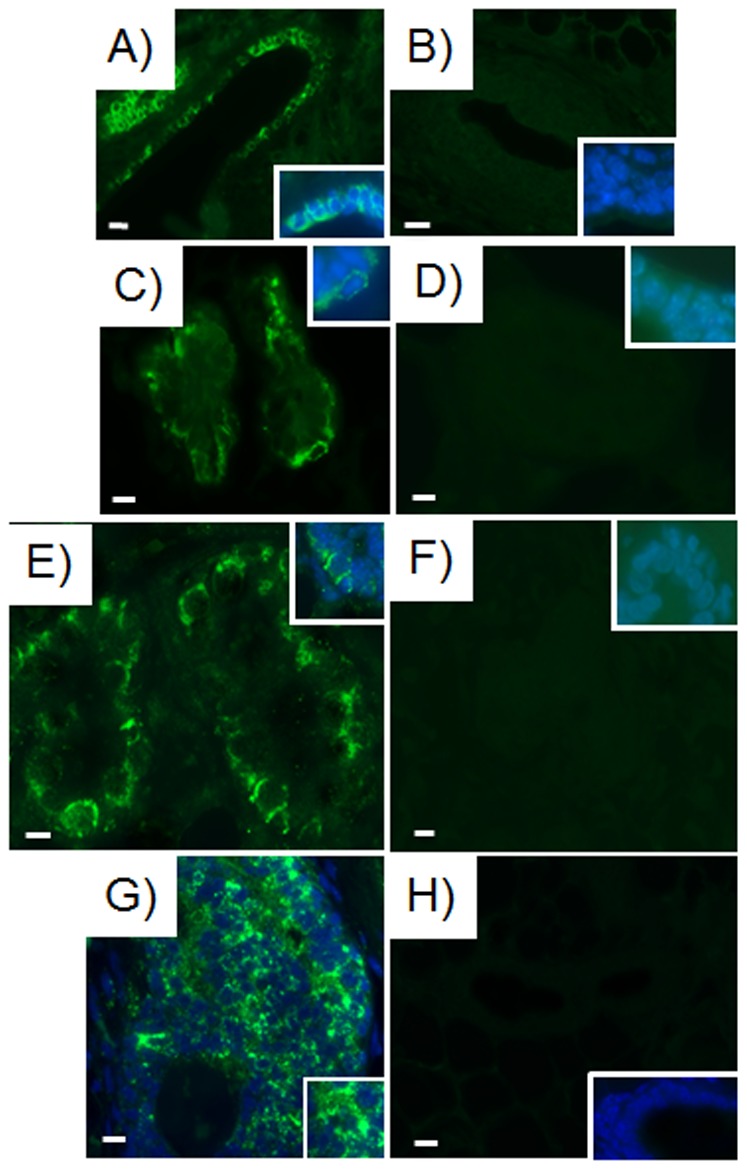
Neither human or mouse specific antibodies were cross-reactive. Human-specific keratin 8 reacts with A) human tissue, but not B) mouse tissue. Human-specific keratin 14 reacts with C) human tissue, but not D) mouse tissue. Mouse-specific keratin 14 reacts with E) mouse tissue but not F) human tissue. Human-specific mitochondria reacts with G) human tissue, but not H) mouse tissue. Normal human breast tissue was obtained from a female undergoing reduction mammoplasty with no evidence of breast disease. Normal mouse mammary tissue was obtained from the untreated, intact abdominal mammary gland of a 15-week-old female athymic nude mouse. All insets, magnification of representative areas with merge of Alexa Fluor 488 (green), FITC (green), or rhodamine (red) plus DAPI (blue). Scale bars 40 µm.

Human breast cancer cells integrated in chimeric outgrowths expressed the human mammary luminal marker keratin 8 (hK8) (Figure 3a–d, Figure S3). Of the 100 cells counted in three separate outgrowths from both primary and secondary transplant generations, hK8 was expressed in 2 out of 5 luminal cells (Figure 3a–d, Figure S3). Human breast cancer cell progeny also expressed the myoepithelial cell marker human keratin 14 (hK14) (1 in 10 cells) and were located adjacent to mouse mammary epithelial cells expressing mouse keratin 14 (Figure 4). When assayed alone in-vitro, MDA-MB-231 cells moderately expressed hK8, but did not express hK14. MDA-MB-231BRMS cells did not express either human keratin 8 or human keratin 14. MDA-MB-468 cells expressed negligible amounts of hK8, but no hK14. hTERT-HME1 cells expressed both hK8 and hK14 (data not shown). Neither human- nor mouse-specific antibodies were cross-reactive with mouse or human epithelium, respectively (Figure 5). These results indicate that human non-metastatic, metastasis-suppressed, and metastatic breast cancer cells contribute to the formation of both luminal as well as basal mammary cells in the regenerated chimeric mammary gland. In addition, these results indicate that cells derived from the original inoculated human breast cancer cell population are capable of proliferation in the regenerated mammary gland and contribute to mammary epithelial cell progeny that differentiate in both the original and second transplant outgrowths. It was estimated by human specific keratin expression that the number of human cells present in the chimeric mammary gland outgrowth was two to five times more than the quantity of human breast cancer cells originally inoculated.

### Human breast cancer cells secrete human milk proteins into the lumen of lactating hosts

The mammary gland undergoes extensive alveolar proliferation and differentiation during pregnancy, resulting in the formation of a complex network of lobules with luminal cells that secrete milk proteins and ducts that carry milk from lobules to the nipple [22]. In order to determine if human breast cancer cell progeny were capable secreting milk proteins, chimeric mammary outgrowths were harvested following a full term pregnancy at day 2 of lactation where human-specific milk protein alpha-lactalbumin was detected (Figure 3e). Human alpha-lactalbumin was not found in sections from intact lactating axillary mammary glands of recipient hosts (Figure 3f). Antibodies directed towards total mouse caseins detected mouse-specific milk protein staining in the secretory lumen of both chimeric mammary gland outgrowths (Figure 3g), and intact axillary mammary glands from lactating host recipients (Figure 3h).

### In-vitro propagation of breast cancer-initiating cells

**Figure 6:**
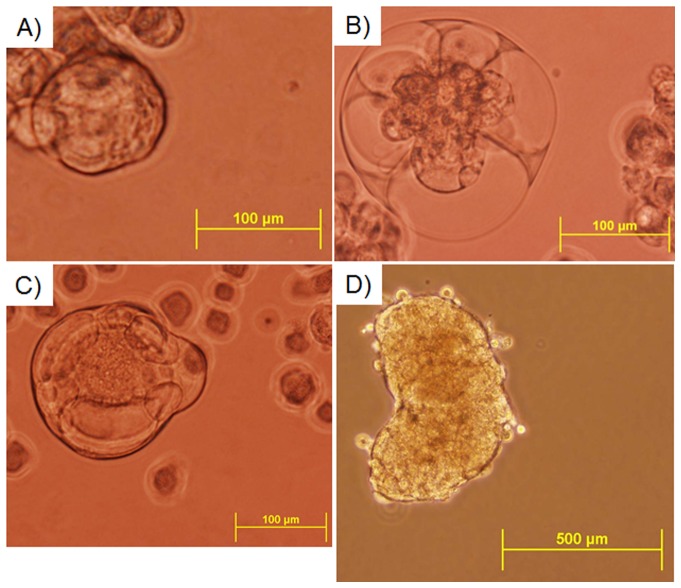
Human breast cancer cells form tumorspheres, which can be propagated after dissociation. Breast cancer cells pre-implantation were cultured under non-adherent conditions in order to elicit the formation of tumorspheres via the tumor-initiating cells in each population. Upon dissociation by enzymatic digestion, tumorspheres were propagated up to three passages. A) MDA-MB-468; B) MDA-MB-231-GFP; C) MDA-MB-231BRMS-GFP; D) hTERT-HME1 cells. All representative images. Scale bars 100–500 µm.

Recent published data have suggested that tumors are composed of a heterogeneous population of cells which consist of, among others, tumor-initiating cells [23]–[25]. Found in a variety of cancers [26]–[30] and identified by the marker CD44, these tumor-initiating cells can be sorted and propagated as non-adherent tumorspheres in suspension culture [21], [31]. MDA-MB-231-GFP, MDA-MB-231BRMS-GFP, and MDA-MB-468 human breast cancer cells were grown under non-adherent conditions and enriched for tumor-initiating cells to determine the presence of tumorsphere-forming tumor-initiating cells. The same populations were enumerated and magnetically sorted into CD44-enriched and CD44-depleted cell fractions and grown under non-adherent conditions. Three to 5% MDA-MB-231-GFP, 10–11% MDA-MB-231BRMS-GFP, and 50–70% of MDA-MB-468 human breast cancer cell parental populations were sorted into CD44-positive fractions. Tumorspheres (≥50 µm in diameter) formed in both the parental, CD44-enriched, and CD44-depleted populations within ∼10 days (parental: Figure 6a–c, CD44-enriched: Figure S4a–c, CD44-depleted: Figure S5a–c). In serial passages, tumorspheres formed for at least four passages when generated using the parental and CD44-enriched cell populations. On the other hand, tumorspheres generated from the CD44-depleted population ceased to grow after one passage. This indicated that the tumor-initiating, self-renewing cancer cells were concentrated in the CD44-positive fraction. As a control, hTERT-HME1 cells were also assessed for their ability to form spheres (termed “mammospheres”). Approximately 60–70% hTERT-HME1 parental cell population was CD44-positive, and mammospheres formed in both the parental and CD44-enriched populations reaching sizes of ≤500 µm (Figure 6d, Figure S4d). Mammospheres rarely formed in populations of cells depleted for CD44 (Figure S5d). Primary hTERT-HME1 mammospheres were sheared with a pipette, enzymatically dissociated to single cells, and passaged. Mammospheres initially formed, however by 15 days post-passage, all the hTERT-HME1 cells were dead.

### CD44-positive Breast Cancer Cells and Mouse/Human Mammary Chimera Formation

**Figure 7:**
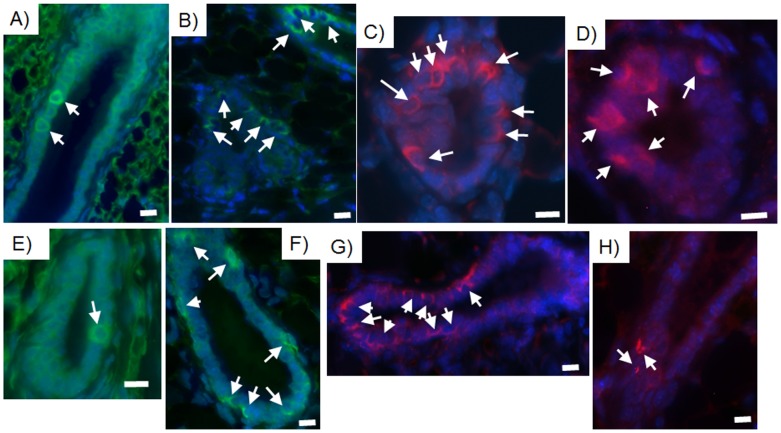
A population of human breast cancer cells expresses markers for human keratin 8, human keratin 14, and human mitochondria in chimeric mammary outgrowths. MDA-MB-231-GFP human breast cancer cells were magnetically sorted for CD44, then separated into CD44-enriched and CD44-depleted populations. Ten thousand of either CD44-enriched or CD44-depleted breast cancer cells were mixed with fifty thousand mouse mammary epithelial cells and inoculated into the epithelium-divested fat pads of three-week-old female athymic nude mice. Twelve weeks later, fat pad outgrowths were harvested and sections made for immunochemistry. Sections were stained for the human-specific luminal epithelial cell marker human keratin 8, the human-specific myoepithelial cell marker human keratin 14, the mouse-specific myoepithelial cell marker mouse keratin 14, and the human-specific marker human mitochondria. A,E) Human keratin 8 (green, Alexa Fluor 488); B, F) human keratin 14 (green, Alexa Fluor 488); C, G) mouse keratin 14 (red, rhodamine); D, H) human mitochondria (red, rhodamine). A–D) CD44-enriched; E–H) CD44-depleted. All outgrowths are first generation chimeras (human breast cancer cells plus mouse mammary epithelial cells). All are representative images. Scale bars 10 µm.

To functionally assess the role of tumor-initiating capabilities of the cell populations in the formation of mammary chimeras, 10,000 CD44-enriched or 10,000 CD44-depleted human breast cancer cells were mixed with 50,000 mouse mammary epithelial cells and implanted into the epithelium divested fat pads of three-week-old female athymic nude mice. Twelve weeks later, mice were euthanized, mammary gland outgrowths harvested and stained for hK8, hK14, mouse keratin 14, and human mitochondria. HK8, hK14, and human mitochondria expression was detected throughout the outgrowths of CD44-enriched implants (∼1 in 7 to 1 in 20 cells)(Figure 7a,b,d). Of the 100 cells counted in three separate outgrowths, cells expressing mouse keratin 14 were found adjacent to cells expressing hK14 in the outgrowths of CD44-enriched implants (∼1 in 5 cells)(Figure 7c). Cells expressing hK8, hK14, and human mitochondria were found in outgrowths from CD44-depleted implants, however these were more rare (∼1 in 30 to 1 in 45 cells)(Figure 7e,f,h). Cells expressing mouse keratin 14 were also detected in outgrowths from CD44-depleted implants (∼1 in 7 cells)(Figure 7g).

### Human breast cancer cells and host mouse epithelial cells do not fuse during mammary gland regeneration

To determine if human breast cancer cells and host mouse mammary epithelial cells fused during mammary gland regeneration, metaphase nuclei were made from cells isolated from second-generation chimeric glands and examined by fluorescent in-situ hybridization (FISH) using human or mouse telomere-specific x chromosome probes. FISH of cells from second generation chimeric outgrowths demonstrated that metaphase spread preparations contained *only* the human telomere-specific x chromosome probe *or* the mouse x chromosome telomere-specific probe (never both). Human (Figure S6a) and mouse (Figure S6b) chromosomes can be easily distinguished by morphology, and none of the metaphase spreads exhibited evidence of fusion between mouse and human cells. Approximately 1 in 15 to 1 in 25 human breast cancer cells were found amongst mouse cells as determined by enumerating the total number of cells in a 10× field of metaphase spreads. Cells with only human chromosomes (n = 5 each, breast cancer cells) contained between 60 to 68 chromosomes (hTERT-HME1 cells: 46–47 chromosomes, n = 5) (Figure S6c) versus mouse cells (n = 4), which contained 38–41 chromosomes (Figure S6d). These numbers corresponded with the number of chromosomes found in human breast cancer cells pre-implantation (n = 5 each, 58–64 chromosomes; hTERT-HME1 cells: 45–50 chromosomes, n = 5; mouse mammary epithelial cells: 38–42 chromosomes, n = 4) (Figure S6e–f).

## Discussion

Previously, we showed that pluripotent human embryonal carcinoma cells could be redirected from their tumorigenic phenotype to differentiation into *functional* bona fide human-specific mammary epithelial cells through interaction with the mouse mammary microenvironment in-vivo [7]. These data suggested that other human cancer cells might also be able to respond to signals in the developing mammary gland. In order to investigate this hypothesis, human metastatic, non-metastatic, and metastasis-suppressed breast cancer cells were mixed with primary mammary epithelial cells and inoculated into the epithelium divested fat pads of three-week-old female athymic nude mice. It was found that human breast cancer cells proliferate and contribute to normal mammary gland development *without* tumor formation in-vivo. When human MDA-MB-231-GFP metastatic, MDA-MB-231BRMS-GFP metastasis-suppressed, and MDA-MB-468 non-metastatic breast cancer cells were mixed with mouse mammary epithelial cells and inoculated into mammary fat pads of mice cleared of epithelium, no primary tumors formed after 6 months, and the human breast cancer cells contributed to the formation of the regenerated mouse mammary gland, differentiated down two distinctly different mammary epithelial pathways (luminal and myoepithelial), and secreted human-specific milk proteins into the lumen of lactating hosts. Furthermore, there was no evidence of human/mouse cell fusion in the chimeric outgrowths as determined by FISH, further suggesting that the human breast cancer cells proliferated independently in the formation of primary and secondary chimeras.

MDA-MB-231 human breast cancer cells have previously been found to express mixed staining patterns of human keratin 18 (luminal cell marker) and human keratin 14 (myoepithelial cell marker) [32]. Under conditions of the experiment described here, MDA-MB-231 cells alone, pre-implantation, expressed moderate amounts of human keratin 8 (luminal cell marker), but no human keratin 14. These observations are in agreement with the staining patterns observed in Figures 4 and 7. It is also possible, however, that MDA-MB-231 cells expressing human keratin 14 may be found in luminal positions, since these cells sometimes express human keratin 14 in-vitro [32].

Due to a lack of expression of the estrogen receptor or progesterone receptor, low mRNA levels of human prolactin receptor (hPRLR), or overexpression of human epidermal growth factor receptor (HER2) [33], it was surprising that human breast cancer cells gave rise to luminal cell progeny capable of producing milk proteins in this study [34]–[35]. Van Keymeulen, et al. showed in the mouse that basal cells (as determined by keratin expression) can adopt a multipotent progenitor fate and completely recapitulate the mammary gland upon transplantation, suggesting a possible mechanism by which the triple negative human breast cancer cells used here might generate mammary gland outgrowths containing hK8-expressing luminal cells [36]. This work also argued that multiple cell types including differentiated mammary cells could contribute to outgrowths generated from cellular injections [36]. Furthermore, it was recently shown that expression of the hPRLR could be restored/enhanced in MDA-MB-231 breast cancer cells through the TGF-beta/Smad and MAP kinase/ERK1/2 signaling pathways [37]. In addition, restoration of hPRLR expression in MDA-MB-231 human breast cancer cells resulted in the cells being less aggressive, less invasive, and influenced the maintenance of an epithelial phenotype [37]. Our observations provide support that human breast cancer cells are capable of redirection from their basal/tumorigenic phenotype towards a ‘normal’ function by the tissue microenvironment.

To determine if the tumor-initiating population of human breast cancer cells was enriched for reprogrammable cancer cells, we selectively isolated breast cancer cells with surface markers (CD44) associated with tumor-initiating activity. In this role, CD44 facilitates tumor progression through the MAP kinase and PI3 kinase/AKT signaling cascades, which subsequently increase cancer cell invasion, growth, motility, and survival [38]. Breast cancer cell enrichment for CD44 did not significantly affect the capacity of the tumor cells to be reprogrammed; however proliferation in-vivo appears to be decreased in the CD44-depleted chimeras. Both CD44-enriched and CD44-depleted cancer cell populations contained cells that could contribute to mouse/human chimeric mammary outgrowths. The number of redirected human breast cancer cell progeny was greater in those chimeras produced with CD44-enriched breast cancer cells; however this result may indicate that tumor-initiating breast cancer cells are more likely to be reprogrammed or alternatively that CD44 reprogrammed breast cancer cells are more likely to produce proliferatively active, reprogrammed progeny. Regardless, these results support our observations that human cells expressing human keratins are present in human/mouse mammary chimeras. Furthermore, differences in the number of detectable human breast cancer cells in CD44-enriched versus CD44-depleted chimeras indicates that our detection of human cells by keratin expression is human-specific, with no cross-reactivity to mouse. To our knowledge, this is the first demonstration that human breast cancer cells are capable of recognizing signals generated by the mouse mammary gland microenvironment during gland regeneration in-vivo. The results suggest there is significant amount of crosstalk between the tissue microenvironment and cancer cells, as well as demonstrates that breast cancer cells can respond to “normal” developmental cues. In addition, these results suggest the dominance of a “normal” microenvironment over tumor development and the possible control or “normalization” of cancer cells in-situ by exposure to factors produced by normal tissues.

## Supporting Information

Figure S1 **Human breast cancer cells contribute to the formation of the mouse mammary gland.** Whole mounts of chimeric mammary gland outgrowths (A–D) were formed from inoculation of human breast cancer cells plus mammary epithelial cells. Twelve weeks post-implantation, mammary gland outgrowths were harvested, fixed in Carnoy's fixative, and stained overnight with Carmine Alum. First generation whole mount of chimeric mammary outgrowth produced from inoculation of A) 1 K MDA-MB-468 plus 50 K mammary epithelial cells; B) 10 K MDA-MB-231-GFP plus 50 K mammary epithelial cells; C) 1 K MDA-MB-231BRMS-GFP plus 50 K mammary epithelial cells; D) 10 K hTERT-HME1 plus 50 K mammary epithelial cells. All outgrowths are first generation chimeras (human breast cancer/human mammary epithelial cells plus mouse mammary epithelial cells). Portions were removed for second transplant generation. Scale bars 1000 µm.
(TIF)

Figure S2 **Human breast cancer cells are present in chimeric mammary outgrowths.** Human breast cancer cells incorporated into the mammary gland and express human-specific mitochondria as indicated by arrows (A–D) (green; Alexa Fluor 488). Human specific mitochondria is expressed in chimeric mammary gland outgrowths produced with A) 1 K MDA-MB-468 plus 50 K mammary epithelial cells, B) 10 K MDA-MB-231-GFP plus 50 K mammary epithelial cells, C) 1 K MDA-MB-231BRMS-GFP plus 50 K mammary epithelial cells, D) 10 K hTERT-HME1 plus 50 K mammary epithelial cells. All outgrowths are first generation chimeras (human breast cancer/human mammary epithelial cells plus mouse mammary epithelial cells). Scale bars 40 µm.
(TIF)

Figure S3 **Human breast cancer cells contribute to the formation of luminal epithelial cells in the chimeric mammary gland.** Human breast cancer cells incorporated into the mammary gland express human-specific luminal epithelial cell marker keratin 8 (arrows, green; Alexa Fluor 488). Human-specific luminal epithelial cell marker keratin 8 (arrows) is expressed in chimeric mammary gland outgrowths produced with A) 1 K MDA-MB-468 plus 50 K mammary epithelial cells, B) 10 K MDA-MB-231-GFP plus 50 K mammary epithelial cells, C) 1 K MDA-MB-231BRMS-GFP plus 50 K mammary epithelial cells, D) 10 K hTERT-HME1 plus 50 K mammary epithelial cells. All outgrowths are first generation chimeras (human breast cancer/human mammary epithelial cells plus mouse mammary epithelial cells). Scale bars 10 µm.
(TIF)

Figure S4 **A population of CD44-enriched human breast cancer cells forms tumorspheres, which can be propagated after dissociation.** Breast cancer cells pre-implantation were magnetically enriched for CD44, then cultured under non-adherent conditions in order to elicit the formation of tumorspheres via the tumor-initiating cells in each CD44-enriched population. Upon dissociation by enzymatic digestion, tumorspheres were propagated up to three passages. A) MDA-MB-468; B) MDA-MB-231-GFP; C) MDA-MB-231BRMS-GFP; D) hTERT-HME1 CD44-enriched cells. All representative images. Scale bars 100–200 µm.
(TIF)

Figure S5 **A population of CD44-depleted human breast cancer cells forms tumorspheres, which can be propagated after dissociation.** Breast cancer cells pre-implantation were magnetically depleted for CD44, then cultured under non-adherent conditions in order to elicit the formation of tumorspheres via the tumor-initiating cells in each CD44-depleted population. Upon dissociation by enzymatic digestion, tumorspheres were propagated up to three passages. A) MDA-MB-468; B) MDA-MB-231-GFP; C) MDA-MB-231BRMS-GFP; D) hTERT-HME1 CD44-depleted cells. All representative images. Scale bars 100 µm.
(TIF)

Figure S6 **Human breast cancer and mouse mammary epithelial cells did not fuse during mammary gland regeneration.** FISH chromosome painting of the human and mouse specific X chromosome and metaphase spreads of cells pre- and post-transplantation. A) Human breast cancer cells prior to transplantation showing human specific X chromosome staining (orange). Three orange signals are seen due to a translocation of an X chromosome. One normal X chromosome can be seen (large arrow) as well as an X chromosome that has undergone a translocation (two smaller arrows). B) Epithelial cells from a chimeric outgrowth containing only mouse chromosomes. Metaphase spreads of C) epithelial cell from chimeric outgrowth containing only human chromosomes and D) epithelial cells from chimeric outgrowth containing only mouse chromosomes. E) MDA-MB-231-GFP cell chromosomes prior to transplantation and F) mouse mammary epithelial cell chromosomes prior to transplantation. No evidence of human/mouse cell fusion was observed and all cells had the expected number of chromosomes. Scale bars 10 µm.
(TIF)

Table S1 **Human breast cancer cells are incorporated in the mouse mammary gland.** Ten thousand MDA-MB-231-GFP human breast cancer cells or hTERT-HME1 human mammary epithelial cells, or one thousand MDA-MB-231BRMS-GFP metastasis-suppressed breast cancer cells or MDA-MB-468 non-metastatic human breast cancer cells were mixed with fifty thousand mouse mammary epithelial cells and inoculated into the epithelium divested fat pads of three-week-old female athymic nude mice. Twelve weeks later, fat pad outgrowths were harvested, and either fragments taken for use in second generation implants, or tissues made into whole mounts, and subsequently sectioned for immunochemistry. Whole mounts were examined for the presence of gland regeneration and tumor formation, and sections stained for the presence of both human- and mouse-specific proteins. Mammary gland outgrowths formed at least 50% of the time with no tumor formation and human cancer cells were present in 100% of sections assayed. Ten thousand MDA-MB-231BRMS-GFP metastasis-suppressed human breast cancer cells or ten thousand MDA-MB-468 non-metastatic human breast cancer cells mixed with fifty thousand mouse mammary epithelial cells and inoculated into the epithelium divested fat pads of three-week-old female athymic nude mice resulted in the formation of tumors, and thus these combinations were not used for future study.
(TIF)
