# Supplementary figures and images for: Human Breast Cancer Cells Are Redirected to Mammary Epithelial Cells upon Interaction with the Regenerating Mammary Gland Microenvironment In-Vivo
Source: PLoS One. 2012 Nov 14;7(11):e49221. doi: 10.1371/journal.pone.0049221 (PMC3498340; doi:10.1371/journal.pone.0049221)

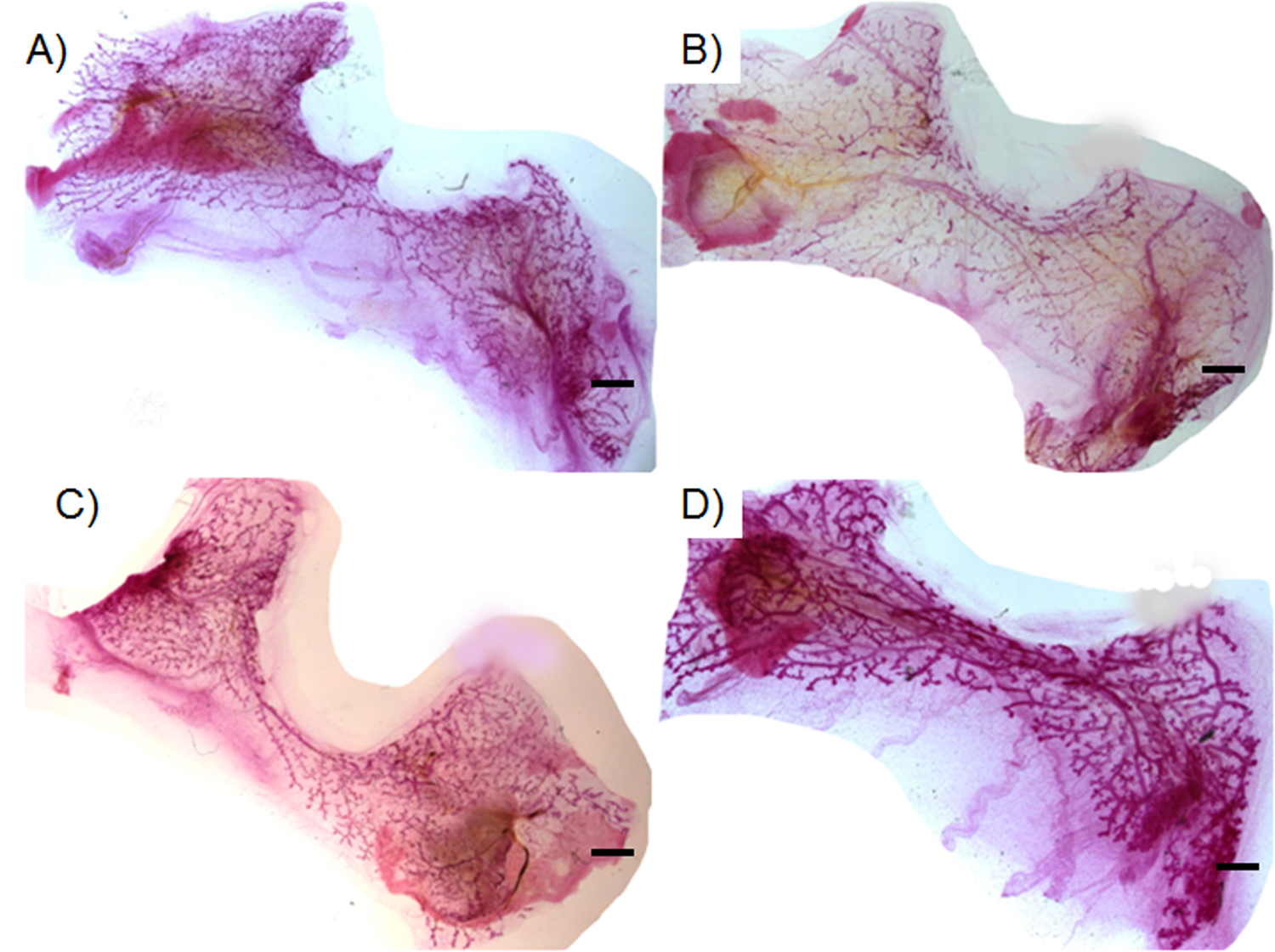

Supplement: Figure S1 — Human breast cancer cells contribute to the formation of the mouse mammary gland. Whole mounts of chimeric mammary gland outgrowths (A–D) were formed from inoculation of human breast cancer cells plus mammary epithelial cells. Twelve weeks post-implantation, mammary gland outgrowths were harvested, fixed in Carnoy's fixative, and stained overnight with Carmine Alum. First generation whole mount of chimeric mammary outgrowth produced from inoculation of A) 1 K MDA-MB-468 plus 50 K mammary epithelial cells; B) 10 K MDA-MB-231-GFP plus 50 K mammary epithelial cells; C) 1 K MDA-MB-231BRMS-GFP plus 50 K mammary epithelial cells; D) 10 K hTERT-HME1 plus 50 K mammary epithelial cells. All outgrowths are first generation chimeras (human breast cancer/human mammary epithelial cells plus mouse mammary epithelial cells). Portions were removed for second transplant generation. Scale bars 1000 µm. (TIF) [file pone.0049221.s001.tif]

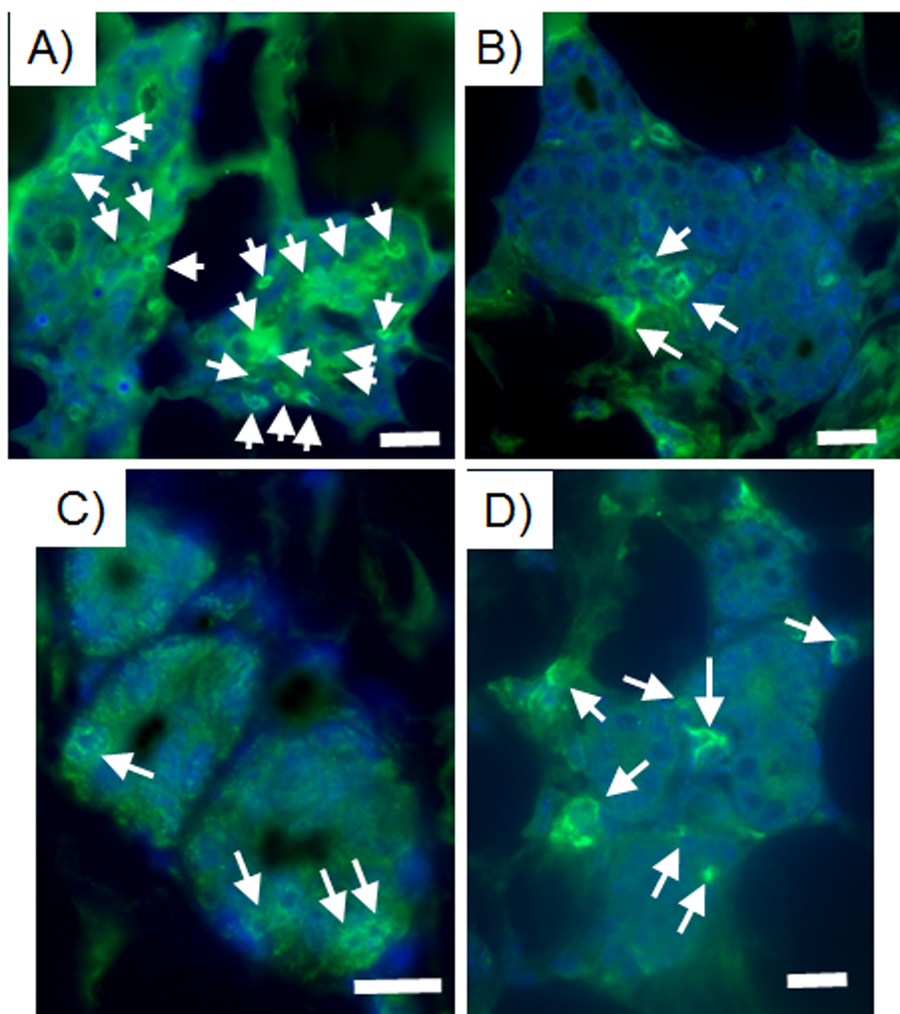

Supplement: Figure S2 — Human breast cancer cells are present in chimeric mammary outgrowths. Human breast cancer cells incorporated into the mammary gland and express human-specific mitochondria as indicated by arrows (A–D) (green; Alexa Fluor 488). Human specific mitochondria is expressed in chimeric mammary gland outgrowths produced with A) 1 K MDA-MB-468 plus 50 K mammary epithelial cells, B) 10 K MDA-MB-231-GFP plus 50 K mammary epithelial cells, C) 1 K MDA-MB-231BRMS-GFP plus 50 K mammary epithelial cells, D) 10 K hTERT-HME1 plus 50 K mammary epithelial cells. All outgrowths are first generation chimeras (human breast cancer/human mammary epithelial cells plus mouse mammary epithelial cells). Scale bars 40 µm. (TIF) [file pone.0049221.s002.tif]

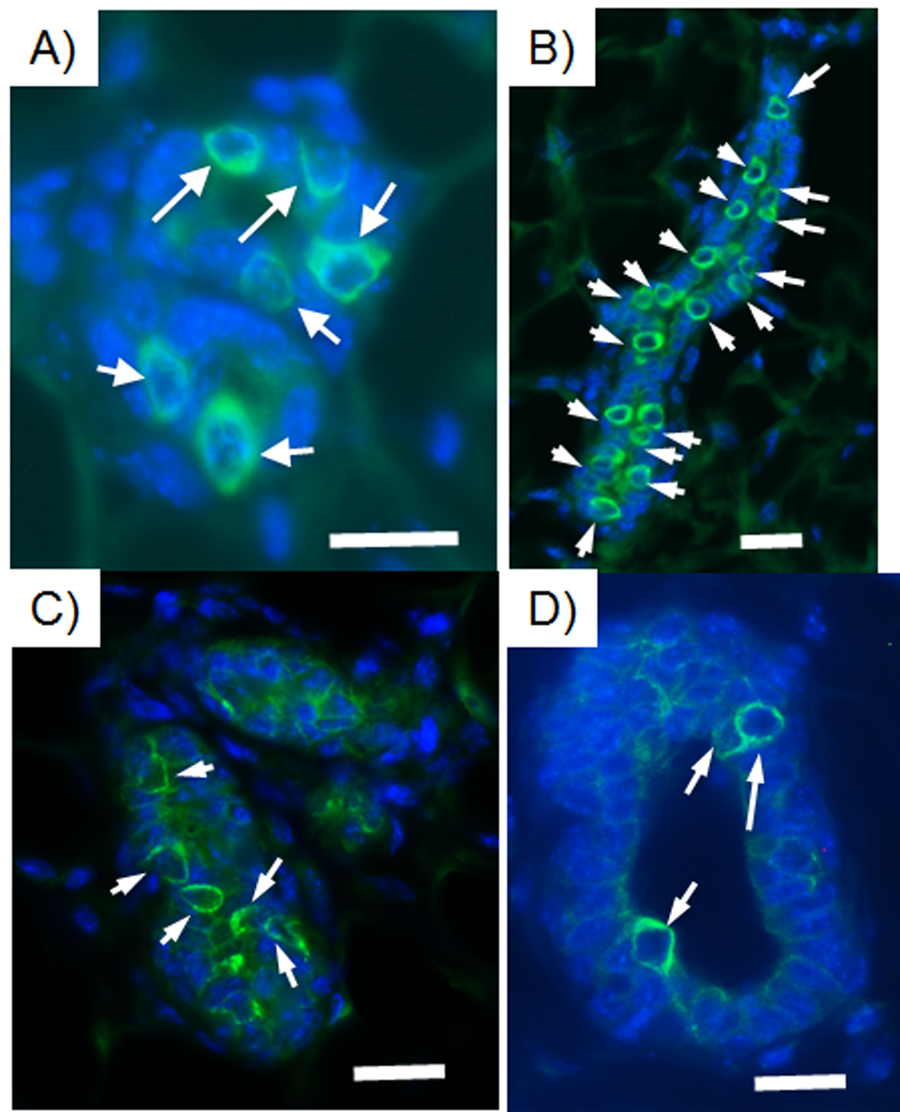

Supplement: Figure S3 — Human breast cancer cells contribute to the formation of luminal epithelial cells in the chimeric mammary gland. Human breast cancer cells incorporated into the mammary gland express human-specific luminal epithelial cell marker keratin 8 (arrows, green; Alexa Fluor 488). Human-specific luminal epithelial cell marker keratin 8 (arrows) is expressed in chimeric mammary gland outgrowths produced with A) 1 K MDA-MB-468 plus 50 K mammary epithelial cells, B) 10 K MDA-MB-231-GFP plus 50 K mammary epithelial cells, C) 1 K MDA-MB-231BRMS-GFP plus 50 K mammary epithelial cells, D) 10 K hTERT-HME1 plus 50 K mammary epithelial cells. All outgrowths are first generation chimeras (human breast cancer/human mammary epithelial cells plus mouse mammary epithelial cells). Scale bars 10 µm. (TIF) [file pone.0049221.s003.tif]

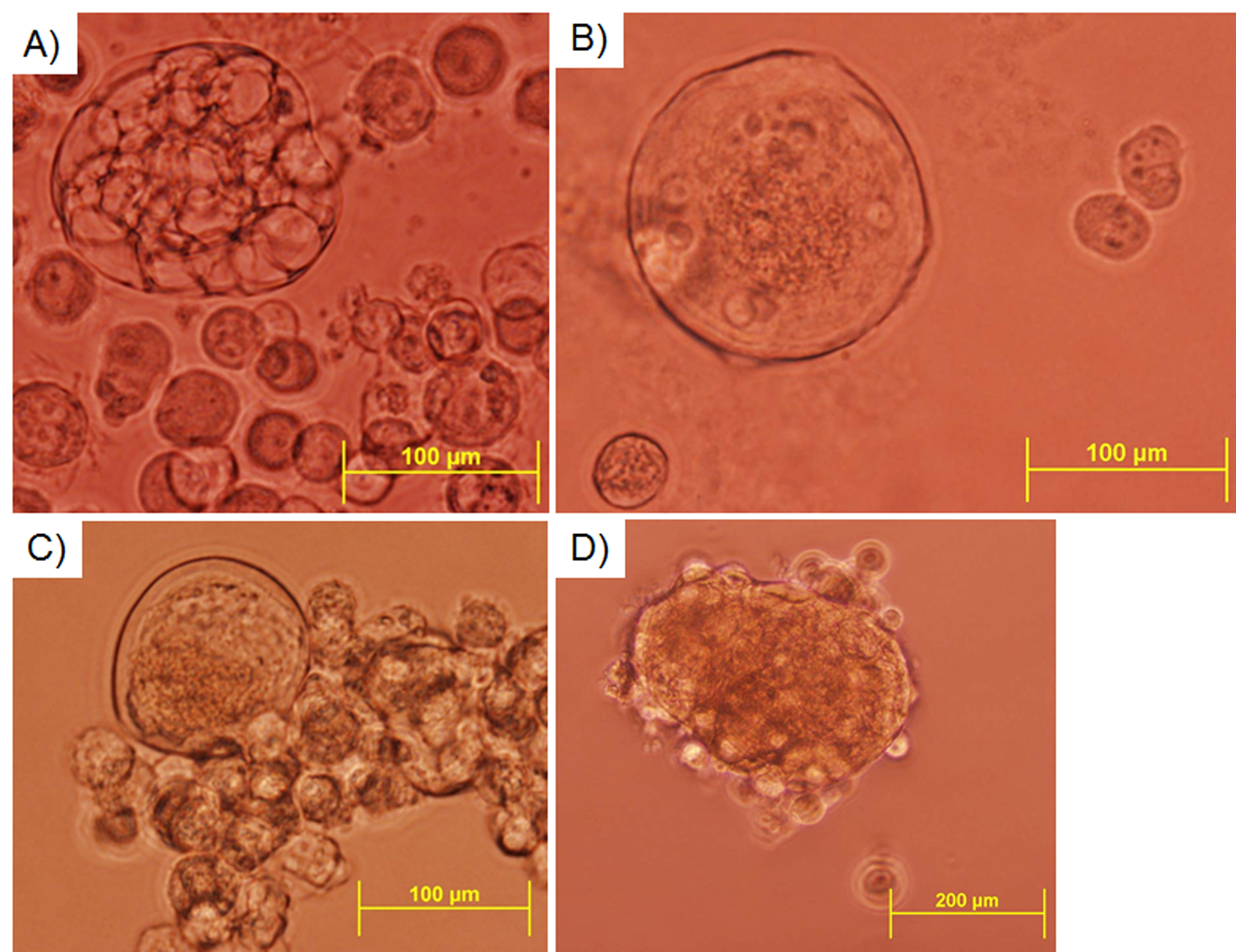

Supplement: Figure S4 — A population of CD44-enriched human breast cancer cells forms tumorspheres, which can be propagated after dissociation. Breast cancer cells pre-implantation were magnetically enriched for CD44, then cultured under non-adherent conditions in order to elicit the formation of tumorspheres via the tumor-initiating cells in each CD44-enriched population. Upon dissociation by enzymatic digestion, tumorspheres were propagated up to three passages. A) MDA-MB-468; B) MDA-MB-231-GFP; C) MDA-MB-231BRMS-GFP; D) hTERT-HME1 CD44-enriched cells. All representative images. Scale bars 100–200 µm. (TIF) [file pone.0049221.s004.tif]

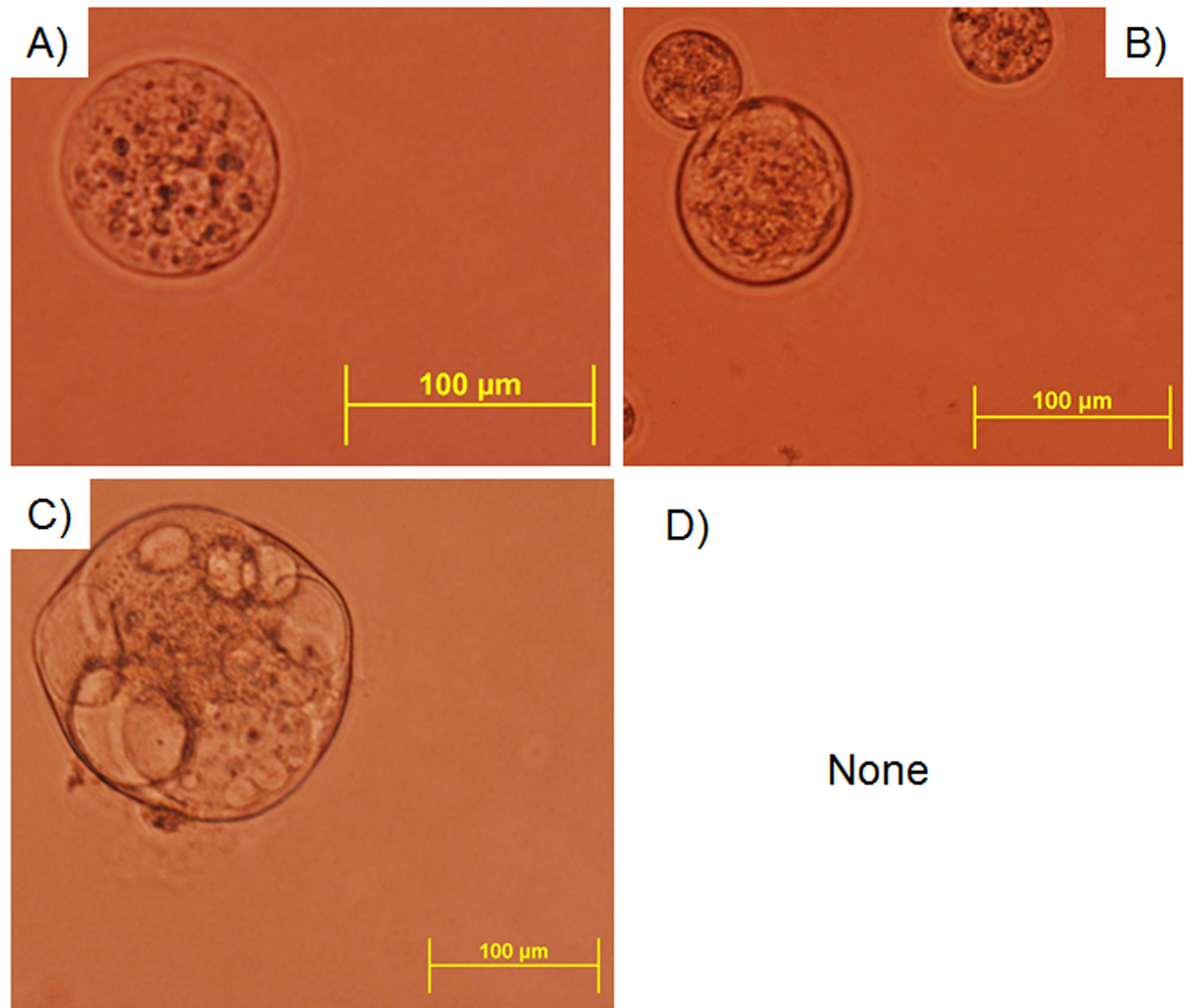

Supplement: Figure S5 — A population of CD44-depleted human breast cancer cells forms tumorspheres, which can be propagated after dissociation. Breast cancer cells pre-implantation were magnetically depleted for CD44, then cultured under non-adherent conditions in order to elicit the formation of tumorspheres via the tumor-initiating cells in each CD44-depleted population. Upon dissociation by enzymatic digestion, tumorspheres were propagated up to three passages. A) MDA-MB-468; B) MDA-MB-231-GFP; C) MDA-MB-231BRMS-GFP; D) hTERT-HME1 CD44-depleted cells. All representative images. Scale bars 100 µm. (TIF) [file pone.0049221.s005.tif]

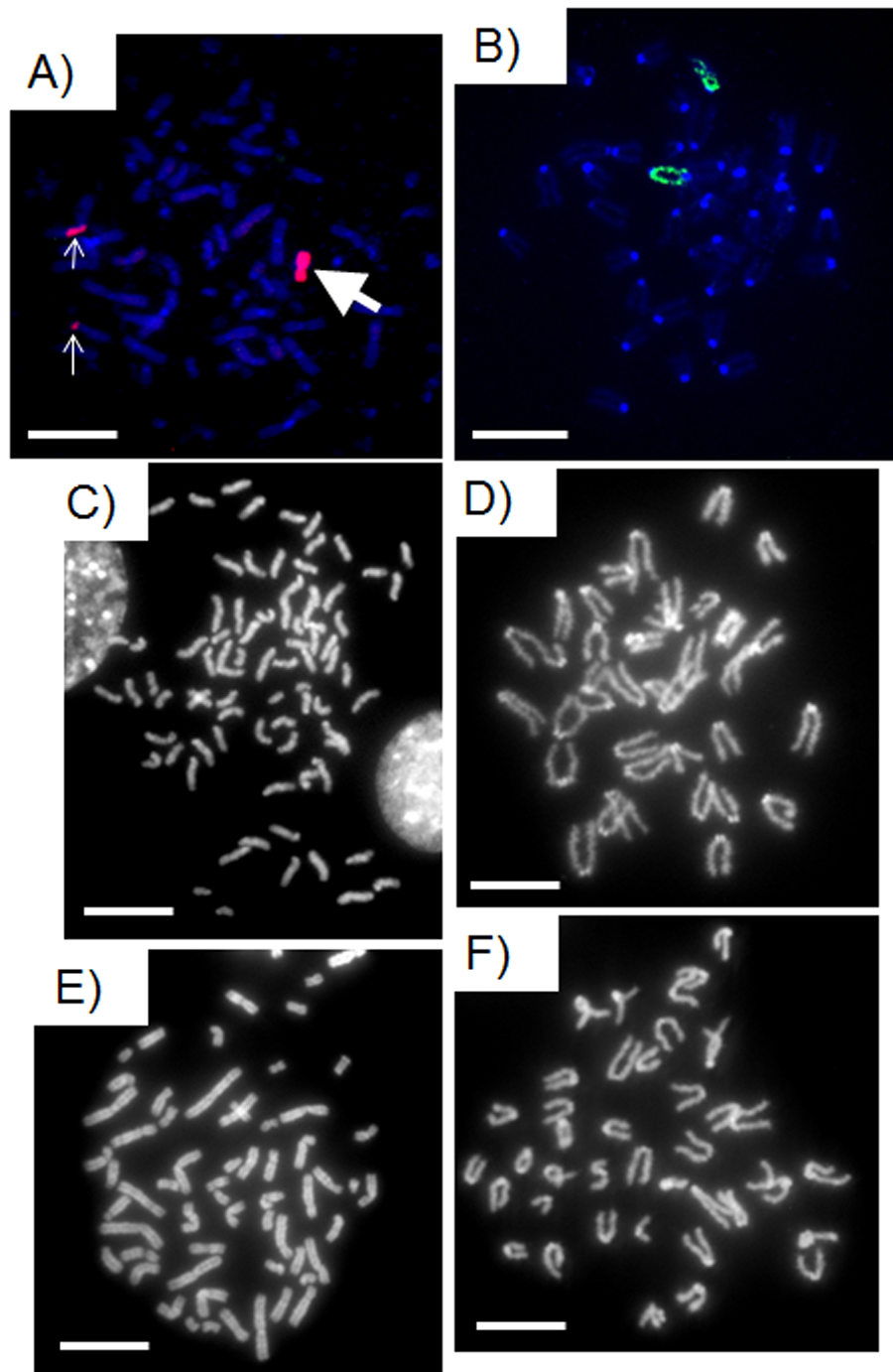

Supplement: Figure S6 — Human breast cancer and mouse mammary epithelial cells did not fuse during mammary gland regeneration. FISH chromosome painting of the human and mouse specific X chromosome and metaphase spreads of cells pre- and post-transplantation. A) Human breast cancer cells prior to transplantation showing human specific X chromosome staining (orange). Three orange signals are seen due to a translocation of an X chromosome. One normal X chromosome can be seen (large arrow) as well as an X chromosome that has undergone a translocation (two smaller arrows). B) Epithelial cells from a chimeric outgrowth containing only mouse chromosomes. Metaphase spreads of C) epithelial cell from chimeric outgrowth containing only human chromosomes and D) epithelial cells from chimeric outgrowth containing only mouse chromosomes. E) MDA-MB-231-GFP cell chromosomes prior to transplantation and F) mouse mammary epithelial cell chromosomes prior to transplantation. No evidence of human/mouse cell fusion was observed and all cells had the expected number of chromosomes. Scale bars 10 µm. (TIF) [file pone.0049221.s006.tif]

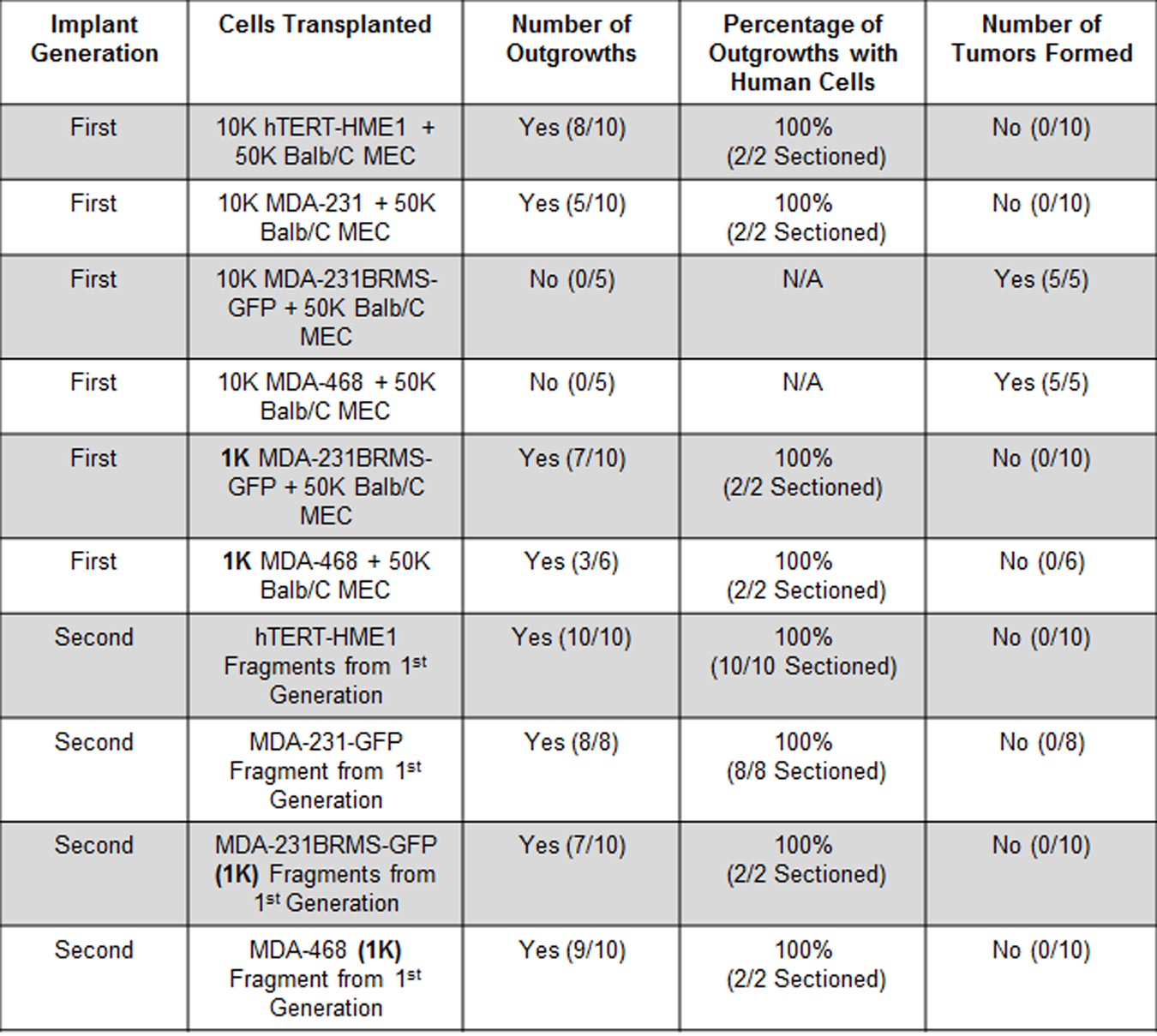

Supplement: Table S1 — Human breast cancer cells are incorporated in the mouse mammary gland. Ten thousand MDA-MB-231-GFP human breast cancer cells or hTERT-HME1 human mammary epithelial cells, or one thousand MDA-MB-231BRMS-GFP metastasis-suppressed breast cancer cells or MDA-MB-468 non-metastatic human breast cancer cells were mixed with fifty thousand mouse mammary epithelial cells and inoculated into the epithelium divested fat pads of three-week-old female athymic nude mice. Twelve weeks later, fat pad outgrowths were harvested, and either fragments taken for use in second generation implants, or tissues made into whole mounts, and subsequently sectioned for immunochemistry. Whole mounts were examined for the presence of gland regeneration and tumor formation, and sections stained for the presence of both human- and mouse-specific proteins. Mammary gland outgrowths formed at least 50% of the time with no tumor formation and human cancer cells were present in 100% of sections assayed. Ten thousand MDA-MB-231BRMS-GFP metastasis-suppressed human breast cancer cells or ten thousand MDA-MB-468 non-metastatic human breast cancer cells mixed with fifty thousand mouse mammary epithelial cells and inoculated into the epithelium divested fat pads of three-week-old female athymic nude mice resulted in the formation of tumors, and thus these combinations were not used for future study. (TIF) [file pone.0049221.s007.tif]
